# Teachers’ Educational Beliefs and Need for Cognition: A Quasi-Experimental Study Based on Philosophical Inquiry

**DOI:** 10.3390/bs16081353

**Published:** 2026-08-06

**Authors:** Fatma Satiroglu Karaagac, Hulya Guvenc

**Affiliations:** 1 Institute of Graduate Studies, Canakkale Onsekiz Mart University, Canakkale 17100, Turkey; 2 Department of Educational Sciences, Faculty of Education, Canakkale Onsekiz Mart University, Canakkale 17100, Turkey; hulyaguvenc@comu.edu.tr

**Keywords:** educational beliefs, need for cognition, philosophical inquiry, educational psychology, teacher

## Abstract

Teachers’ educational beliefs act as psychological frameworks that shape how they interpret educational constructs such as teaching, learning, and classroom practice. Need for cognition (NFC) is associated with motivation in learning, problem-solving, and decision-making processes. This quasi-experimental study used a non-equivalent pretest–posttest control group design to examine the associations of a philosophical inquiry-based professional development programme (P4TEduP) with in-service teachers’ NFC and educational belief subscales. The study included 28 teachers from a single private school; 13 participated in the experimental group and 15 in the control group. The P4TEduP was delivered over five days in 10 sessions and used philosophical inquiry stimuli focused on educational concepts. Data were collected using the Need for Cognition Scale and Educational Belief Scale and analysed using paired-samples t-tests, Wilcoxon signed-rank tests, and a two-stage rank-based residualised ANCOVA with HC3 heteroscedasticity-consistent standard errors. After controlling for pretest scores, no statistically significant between-group differences were found in NFC or educational belief subscales. However, NFC and progressivism increased significantly only within the experimental group. Given the small, non-randomised sample and non-significant baseline-adjusted between-group differences, the findings should be interpreted as preliminary empirical and practical evidence, not as support for programme effectiveness or a new theoretical model.

## 1. Introduction

The increasing ethnic and cultural diversity in schools, changing interests and needs of children, and the evolving societal expectations regarding education are reflections of social change and necessitate a re-evaluation of teachers’ moral obligations (Villegas & Lucas, 2002), skills and competencies. Teachers must critically reflect on their professional practices and classroom positions, interactions, and power relationships with students, pedagogical understandings (Arvaja & Sarja, 2021), and their roles and thinking tendencies (Arvaja, 2016) dialogically and democratically. They should consider the meanings they attach to concepts, such as students, knowledge, learning, teaching, and education.

## 2. Literature Review

### 2.1. Teachers’ Educational Beliefs

Teachers’ educational beliefs act as psychological frameworks for processing knowledge, perceiving learning environments, and justifying classroom actions (Northcote, 2009; Pajares, 1992). In educational psychology, these beliefs are seen as complex, interrelated structures about teaching and learning (Northcote, 2009). Educational beliefs are defined as ‘teachers’ attitudes towards education in general, including their views on school, learning, teaching and students’ (Northcote, 2009; Pajares, 1992). As per Belo et al. (2014), there are three basic assumptions about teacher beliefs that may influence the perpetuation of ineffectual and antiquated teaching approaches. Regarding these three teacher beliefs, first, upon enrolling in teacher education programs and commencing their teaching careers, pre-service teachers possess preconceived notions regarding pedagogy and learning. Second, the beliefs of educators are elements of larger thought systems, which, in turn, encompass views on various topics (e.g., educational goal-setting, the essence of information and its acquisition, and the assessment and management of students’ learning processes). Third, teacher beliefs have a significant association with cognitive monitoring and knowledge interpretation. Thus, teacher beliefs act as perceptual filters, moderating how new information is processed (Belo et al., 2014), and have been posited to be formed through educational philosophy (Livingston et al., 1995; Pajares, 1992). Furthermore, educational philosophy can be used to clarify teachers’ beliefs about the objectives of education, educational assessments, the characteristics of educational content, teaching–learning processes, and the roles of students and teachers (Sahin, 2015). According to Yılmaz et al. (2011), teachers’ and pre-service teachers’ views on these different topics are determinants of the educational beliefs they hold. These aforementioned authors also analysed teachers’ educational beliefs through the frameworks of perennialism, essentialism, progressivism, reconstructionalism, and existentionalism (Yılmaz et al., 2011).

Perennialism, which draws its philosophical foundation from realism, focuses on the immutability of human nature and centers on the authoritative teacher, who transmits universal truths through the Socratic method (Beatty et al., 2009; Demirel, 2015; Ornstein & Hunkins, 2012/2016; Sönmez, 2009). This view foregrounds enduring and universal values; education also focuses on universal values grounded in objective reality and the laws of nature (Beatty et al., 2009). Similarly, essentialism, grounded in idealist and realist foundations, emphasizes basic skills and content rather than students’ interests and positions the teacher as a subject-matter expert and authority (Beatty et al., 2009; Ornstein & Hunkins, 2012/2016).

Progressive educational philosophy, a reform movement in educational, social, and political matters, emerged from pragmatic philosophy (Demirel, 2015; Ornstein & Hunkins, 2012/2016). Progressivism supports education based on problem solving and dialogue, arguing that knowledge changes through interaction with the environment and that values are situational (Beatty et al., 2009; Ornstein & Hunkins, 2012/2016). In addition, reconstructionalism aims at social reform, analyzes power relations and social structures, and views the school as an agent of change (Beatty et al., 2009). Existentionalist educational philosophy is a European philosophy that developed in earlier periods but gained popularity after World War II (Ornstein & Hunkins, 2012/2016). Existentionalism rejects group norms, authority, and the established order, and it is based on the idea that individuals create their own value systems and truths through free choices, personal experiences, and aesthetic preferences (Beatty et al., 2009; Ornstein & Hunkins, 2012/2016). Therefore, existentialists see education as helping students develop their own value systems without adult authority. The existentialist teacher helps the student develop their personality (Demirel, 2015).

Sahin (2015) indicated that progressivism and reconstructionalism are strong predictors of a deep learning strategy among pre-service teachers, while perennialism and reconstructionalism predict a surface learning method. Teachers’ educational beliefs are also associated with their curriculum design approaches (Kozikoğlu & Uygun, 2018) and considered crucial determinants of their professional development (Ashraf & Kafi, 2017).

The main roles of educational philosophy are to question education as a social effort and evaluate the issues affecting educational practices (Mwinzi, 2020). These roles should not be regarded as being independent of teachers’ thinking skills and tendencies. Considering this assertion, we see the need to emphasise the concept of the ‘need for cognition’ (NFC). Gülgöz and Sadowski (1995) address need for cognition in cognitive and educational psychology. They describe it as a motivational construct that explains differences in learning, problem-solving, and decision-making. When mental ability or cognitive strategies do not explain interpersonal differences, need for cognition is considered with willingness, mental effort, and motivation (p. 17).

### 2.2. Need for Cognition

Conceptually, need for cognition highlights a person’s tendency to enjoy and participate in cognitive activities that require effortful thinking (Cacioppo & Petty, 1982), representing a form of intrinsic motivation. The NFC stems from a sense of personal satisfaction, competence, and values, and a force that arises when people face cognitive challenges (Cacioppo et al., 1996). The NFC and the tendency to be satisfied with it indicate individual differences in various domains of learning, including attitudes and beliefs, reasoning and decision-making, interpersonal and group dynamics, and practical applications (Petty et al., 2009). The differences between individuals with low and high NFC usually stem from past experiences, which affect the solution of current problems or dilemmas (Cacioppo et al., 1996). Individuals with high NFC think comprehensively, assume an active role in solving dilemmas, and strive to acquire knowledge, as well as reasoning and problem-solving skills, to cope with dilemmas. While individuals with high NFC enjoy participating in cognitively challenging tasks, individuals with low NFC have low motivation regarding such tasks. Among pre-service teachers and teachers, NFC is associated with their early professional identity (Arpacı & Bardakçı, 2016), problem-solving skills (Polat & Tümkaya, 2010), thinking styles (Uyanık Sağlam & Tunç, 2018), academic achievement (Hawthorne et al., 2021), individual innovativeness (Süer & Kinay, 2019), and teacher burnout (Zerna et al., 2022).

### 2.3. Philosophical Inquiry

Philosophical inquiry offers teachers a teaching method that alleviates some of the negative impacts caused by a prescriptive and goal-oriented educational program, allows for questioning many issues regarding participatory citizenship in a democracy, and assists in the search for humane ways to recognise and assess individual development and contributions (Haynes, 2002, p. 116). In this sense, philosophical inquiry can also be used to examine the meaning of being a teacher and teacher education (Lewis & Robinson, 2017).

The most systematic approach to philosophical inquiry in the literature is ‘Philosophy for Teachers’ (P4T) and can be considered an important guide and tool. P4T includes practices, principles, and methods from the Philosophy for Children (P4C) and aims to engage teachers through philosophical inquiry in a democratic environment over a specified period (Orchard et al., 2016). The philosophical inquiry process present in P4C develops these thinking styles and comprises preparing for inquiry, sharing a stimulus to promote inquiry, time for thinking, inquiry, connecting questions, selecting a question to start the inquiry, building on ideas, recording the discussion, closing and evaluating (Haynes, 2002). It emphasises students asking philosophical questions and developing ideas about related subjects through cooperative talks rather than didactical philosophy teachings. In that philosophical inquiry approach, higher-order thinking development occurs by promoting the interaction between four fundamental thinking styles: critical, creative, cooperative, and caring thinking (Hannam & Echeverria, 2009).

Concurrent with P4C’s function in teacher education, the P4T methodology seeks to generate more meaningful teaching by encouraging teaching practices that go beyond best practices and foster a strong perception of their own educational philosophy. The essential elements of P4T include those outlined hereinafter: encouraging teacher curiosity and dialogue to redefine their own classroom positionality, student roles, and the meaning of learning; reflecting on educational practices, theories, and socially constructed educational norms; affording opportunities for collaborative inquiry in an intellectually safe community; focusing on metacognitive thinking skills development (Bush, 2017, pp. 212–213). Research on the effects of P4T in in-service teacher educational settings indicates that philosophical inquiry, as promoted through P4T, contributes to the development and strengthening of teachers’ pedagogical understanding, instructional practices, and collaborative and reflective thinking skills (Lam, 2021; Roberts, 2006; Scholl, 2014; Scholl et al., 2016). These findings suggest that P4T may influence teachers’ instructional practices and the belief systems that underpin them.

This research examined the association between the implementation of a philosophical inquiry programme, the ‘Philosophy for Teachers Education Program’ (P4TEduP), based on P4T’s main processes, and changes in teachers’ educational beliefs and NFC. Prior P4T studies have generally focused on professional ethics in teacher education (Orchard et al., 2016), which is one of the foundations of P4T. The present programme addressed teachers’ professional ethical dilemmas through several philosophical dimensions—epistemology, political philosophy, educational philosophy, and ethics—thereby providing a broader empirical context for examining P4T in in-service teacher education. Rather than proposing a new theoretical framework, this design was intended to generate preliminary evidence regarding the associations of a philosophical inquiry-based programme with teachers’ NFC and educational beliefs. This approach may help teachers identify their own thinking patterns, critically examine their decision-making processes, and develop stronger reasoning and problem-solving skills. These potential benefits are consistent with Goodman’s (2017) finding that diversity of perspectives significantly predicts need for cognition.

Second, most relevant previous studies were conducted with pre-service teachers. Since the educational beliefs of these teachers mostly reflect their student experiences and theoretical knowledge, the findings of these studies may not provide an optimal take on—and especially not afford optimal accuracy regarding—the effectiveness of P4T in teacher education. Therefore, we assumed that conducting a study with teachers with some experience and more well-established educational beliefs could provide preliminary empirical evidence that complements the existing literature by examining the associations of P4T with relevant variables in an in-service teacher context.

Furthermore, an examination of in-service teacher training programs reveals that these programs emphasise teachers’ curriculum orientation (Bümen & Holmqvist, 2022), along with their adaptation to new teaching (Haapaniemi et al., 2021) and assessment approaches (Wolterinck et al., 2024), methods and technologies (Al-Sinani & Al Taher, 2023). Although these training efforts are crucial and support teacher awareness about new trends in education, teachers who do not undergo radical changes in their educational beliefs may not integrate the novelties into their teaching practices. Bush (2017) proposed the need for a change in the perspective about teaching by encouraging teachers to question and reflect, such that they eventually perceive teaching as a philosophical endeavour. The P4TEduP was proposed following this rationale, and it was expected that the programme would support teachers in reflecting on their educational beliefs.

Importantly, despite the NFC being related to many cognitive and affective characteristics of teachers, no study has hitherto assessed the influence of an in-service teacher educational programme on this variable. Egan et al. (2019) found that the NFC influences primary school teachers’ preferences regarding school-based initiatives for behavioural and emotional issues. Accordingly, the present study examines teachers’ NFC in a professional development context. The purpose of this study was to examine the association of the P4TEduP, a philosophical inquiry-based professional development programme, with in-service teachers’ need for cognition and educational beliefs. Therefore, this study defined two sub-aims described through the following questions:

- What is the association of the P4TEduP with changes in teachers’ NFC after programme implementation?
- What is the association of the P4TEduP with changes in teachers’ educational beliefs after programme implementation?

## 3. Materials and Methods

### 3.1. Research Model

This study used a quasi-experimental pretest–posttest control group design, more specifically a non-equivalent control group design (Creswell, 2014/2017). This design was chosen because it was appropriate for examining the P4TEduP in a natural school-based professional development context where random assignment was not feasible. Including a control group in the design was deemed necessary to provide a preliminary comparison of changes associated with participation in the P4TEduP. It was important for the participating teachers to experience the programme in a face-to-face and collaborative discussion environment so that a community of inquiry could be intellectually and democratically developed. Accordingly, although this study included experimental and control groups, the teachers were not randomly assigned to them. The experimental group received the P4TEduP, whereas the control group did not receive the programme during the main study period. This study was approved by the Canakkale Onsekiz Mart University Scientific Research Ethics Committee (Approval no. E-84026528-050.01.04-2300181837; date: 3 August 2023) and the Ministry of National Education of Turkey.

### 3.2. Participants

This study was conducted within the scope of school-based professional development activities. Therefore, the researchers contacted the administrators of schools that could accept and adapt their activities to incorporate the P4TEduP in-service training programme. The study was conducted in a single school in Istanbul, Turkey, with the school administration’s approval. The school offers education from preschool through high school. In educational research, it is useful for researchers to choose, as their sample, groups of people (students, teachers, and other individuals) with certain characteristics (Fraenkel et al., 2012, p. 92) so that they can spontaneously form groups. Therefore, a non-probability sampling strategy was used because it may be preferable in these research settings.

Teachers at the participating school were informed that participation was voluntary and that they could choose to join either the experimental or control group. Before the study, they received a consent form outlining the study’s purpose, duration, general operations, and rules governing the P4T sessions. Voluntary participation in the P4T sessions was emphasised to help foster an intellectually and socially engaged community of inquiry and to encourage sincere and willing contributions during the sessions. In total, 28 teachers volunteered; 13 joined the experimental group, and 15 joined the control group. See Table 1 for participants’ characteristics.

### 3.3. P4TEduP

The intervention used in this study was the P4TEduP, a philosophical inquiry-based education program developed by the researchers. The first element in a philosophical inquiry-based program is ‘stimuli’, while the second is ensuring that the P4C’s inquiry process is guided by a competent ‘facilitator’.

#### 3.3.1. Philosophical Inquiry Stimuli

Ten philosophical inquiry stimuli were prepared for this programme. These stimuli pertained to philosophical subdisciplines, such as epistemology, political philosophy, educational philosophy, and ethics, and comprised visual-auditory and written media. Of these, seven were designed by the researchers, while the remaining were prepared by an institution that provides P4C training to support researchers who wish to apply the P4TEduP. The three stimuli included an animated film and two written texts pertaining to epistemology and political philosophy, and served to question concepts, such as knowledge, curiosity/inquiry, and education system.

Among the seven stimuli developed by the researchers, six were written texts, and one was a movie pertaining to ethics, education, and political philosophy. Among the text stimuli, two were summaries or sections of the stories ‘Overcoming Obstacles’ (Dağdeviren, 2009, pp. 45–47) and ‘A Little Boy’ (Aydın, 2013, pp. 41–43). The former focused on inquiry into ‘inclusive education/individual differences in education’ and the latter on the concept of ‘teacher’. The movie promoted inquiry into ‘ideal education’. The other self-developed four texts; each addressed one of the following four topics/concepts: professional responsibility; professional competence; benefit in education; and equality and justice in education.

#### 3.3.2. P4TEduP Pilot Study

A pilot study was conducted at a university’s faculty of education. Although 60 pre-service teacher students were invited to participate voluntarily in the pilot study, only 5–15 pre-service teachers participated in the inquiry sessions, as participation was not compulsory. The pilot study activities were conducted online and recorded owing to the COVID-19 pandemic. Later, the practitioner–researcher, who was the researcher implementing the P4TEduP and had completed a 75 h, three-level P4C Trainer Training Specialist Programme provided by a Pearson Assured organisation, and the observer, who was a faculty member in the Department of Educational Sciences with research expertise in cooperative learning, self-regulated learning, teacher motivational support, teachers’ professional self-efficacy, and ethical dilemmas, and who also served as the doctoral supervisor, examined the recordings. After the recordings were reviewed, the practitioner–researcher and observer conducted weekly reflective interviews about the sessions. These interviews focused on the suitability of the stimuli for the targeted philosophical subdisciplines, the clarity and semantic effect of the text-based stimuli, the attractiveness of the stimuli, and the way participants engaged with the concepts during the inquiry process. During the pilot study, first, the suitability of the P4T stimuli to the inquiry process into the targeted sub-philosophy field, as well as their attractiveness, was examined. Special focus was placed on whether the semantic effect of the six text stimuli we developed met the expectations. The reflective interviews informed revisions to the P4T stimuli and helped refine the session structure before the teacher implementation.

#### 3.3.3. Philosophical Inquiry Process

The philosophical processes conducted with teachers within the scope of the P4TEduP included the following: 1. Preparation for Inquiry: explaining the rules and processes to the community of inquiry, 2. Sharing the Stimulus: presenting and summarising the stimulus to the community, 3. Community of Inquiry: participants thinking about questions, sharing their thoughts and deeply questioning the stimulus, 4. Evaluation: summarising emerging concepts and evaluating the process and 5. Sharing information about the stimuli (i.e., sources, questioned concepts and philosophical subdisciplines). These stages were used to structure the philosophical inquiry process rather than to function as separate assessments of learning outcomes. The programme consisted of 10 sessions, with two sessions conducted each day for five consecutive days. In the first session of each day, we systematically implemented all five stages. Throughout all the second sessions, we omitted the ‘Preparation for Inquiry’ stage and proceeded directly to the remaining four stages. This adjustment, built on the groundwork from earlier sessions, improved time management. Each session lasted 50–55 min. After the first session, there was a 30 min break, which was followed by the second session. Including the break, the total duration of the programme each day was approximately 130–140 min. A researcher, trained in the P4C Trainer Training Specialist Program (75 h, three levels) provided by a Pearson Assured organisation (P4C Turkey, n.d.) in 2021–2022, implemented the programme. The same researcher was involved in developing the P4TEduP and facilitated all sessions in accordance with the predetermined session structure and inquiry stages described above. All teachers in the experimental group attended all P4TEduP sessions.

### 3.4. Measurements

Various measures were used to assess the association between programme implementation and changes in outcome variables. Participants completed the questionnaire in approximately 25 min.

#### 3.4.1. Educational Belief Scale

The Educational Belief Scale was developed by Yılmaz et al. (2011) to assess teachers’ and prospective teachers’ educational beliefs. The scale comprises 40 items across five subdimensions: progressivism (13 items), existentionalism (7 items), reconstructionalism (7 items), perennialism (8 items), and essentialism (5 items). Responses are provided on a five-point Likert-type scale from 1 (I totally disagree) to 5 (I totally agree). The scale does not yield an overall educational belief score; instead, each subscale is evaluated separately. Higher scores on a given subscale indicate stronger endorsement of the corresponding educational philosophy. The development study involved teachers and prospective teachers, and Cronbach’s alpha values for the subscales in the original study were 0.91, 0.89, 0.81, 0.70, and 0.70 for progressivism, existentionalism, reconstructionalism, perennialism, and essentialism, respectively, demonstrating high reliability (Yılmaz et al., 2011). According to Cronbach’s alpha and McDonald’s omega coefficients, the pretest alpha coefficients were 0.825, 0.877, 0.835, 0.783, and 0.862 for progressivism, existentionalism, reconstructionalism, perennialism, and essentialism, respectively, while the corresponding omega coefficients were 0.802, 0.883, 0.859, 0.803, and 0.872. The posttest alpha coefficients were 0.901, 0.902, 0.882, 0.822, and 0.838, respectively, while the corresponding omega coefficients were 0.831, 0.903, 0.903, 0.816, and 0.857.

#### 3.4.2. NFC Scale

In this study, the short form of the NFC Scale (Cacioppo et al., 1984), adapted into Turkish by Demirci (1998), was used to measure NFC. The original 18-item, single-factor NFC short form is a 5-point Likert-type scale ranging from extremely uncharacteristic of me (1) to extremely characteristic of me (5). Half of the items are worded positively and half are worded negatively. Some examples of scale items are “I would prefer complex to simple problems” and “Thinking is not my idea of fun” (reverse scored). Before calculating the total NFC score, the negatively worded items were reverse-scored, and the item scores were then summed. Higher total scores indicate a higher need for cognition. Demirci reported the test–retest reliability of the scale as 0.89. In various studies, the Cronbach’s alpha reliability coefficient of the scale has been reported as 0.68 to 0.90 (Arpacı & Bardakçı, 2016; Demir & Tunç, 2023; Saracaloğlu & Çengel, 2013; Şener, 2024). For the NFC Scale, the Cronbach’s alpha coefficients were 0.882 at pretest and 0.924 at posttest, while the corresponding McDonald’s omega coefficients were 0.882 and 0.887, respectively.

### 3.5. Data Collection

The program ran from 25 to 29 September 2023. On the first day, before the intervention, the pretest was administered to the experimental and control groups using the Need for Cognition Scale and the Educational Belief Scale. Participants used self-generated codes in both the pretest and posttest so their responses could be matched across measurements. The P4TEduP programme was then implemented with the experimental group in a classroom by one of the researchers, after school hours and following student dismissal. During this period, the control group did not receive the P4TEduP or any alternative intervention. On the last day, a posttest was conducted with both groups using the same instruments. All teachers in the study completed both measurements and were included in the final analysis. After the posttest data had been collected, the programme was offered to the control group from 2 to 6 October 2023, completing the full intervention sequence.

### 3.6. Data Analysis

Data were analysed using IBM SPSS Statistics for Windows, Version 21.0 (IBM Corp., Armonk, NY, USA). Descriptive statistics were performed for the dependent variables. Internal consistency was examined separately for the pretest and posttest using Cronbach’s alpha and McDonald’s omega coefficients.

Skewness and kurtosis coefficients were then calculated to examine the normality of the data. The selection of statistical tests was based on the distributional characteristics of each measured variable and educational belief subscale. For normally distributed variables, within-group comparisons were conducted using paired samples t-tests. For non-normally distributed variables, the Wilcoxon signed-rank test was applied for within-group comparisons. Accordingly, paired samples t-tests were used for the need for cognition scores because the pretest and posttest scores in both groups met the normality criterion. For the educational belief subscales, the choice of parametric or non-parametric tests varied according to the normality results for each subscale and group. The educational belief subscales were analysed separately, as the scale does not yield an overall educational belief score.

To examine between-group differences while controlling for baseline scores, a robust rank-based ANCOVA procedure was conducted using IBM SPSS Statistics. For each outcome, pretest and posttest scores were ranked, and the ranked posttest score was regressed on the ranked pretest score. The resulting residuals were then analyzed in a univariate general linear model with study group as the fixed factor. Type III sums of squares were used, and group effects were tested with HC3 heteroscedasticity-consistent standard errors. Two-sided tests were conducted using a significance level of alpha = 0.05, and 95% confidence intervals were reported. The same procedure was applied separately to NFC and each educational belief subscale. Because separate analyses were conducted for NFC and the five educational belief subscales, the subscale analyses were treated as exploratory and interpreted cautiously without adjustment for multiple comparisons.

Effect sizes were calculated for all reported inferential comparisons to indicate the magnitude of the observed differences, regardless of statistical significance. For paired samples t-tests, Cohen’s d was computed by dividing the t-value by the square root of the sample size. For independent samples t-tests, d was calculated by dividing the difference between group means by the pooled standard deviation. For the Mann–Whitney U and Wilcoxon signed-rank tests, effect size r was calculated by dividing the standardised test statistic Z by the square root of the number of observations; values of 0.5, 0.3, and 0.1 were interpreted as large, moderate, and small effects, respectively (Fritz et al., 2012). For Cohen’s d, values of 0.8 or higher indicated a large effect, 0.5 a moderate effect, and 0.2 or lower a small effect, as conventional thresholds for interpretation (S. B. Green & Salkind, 2005).

## 4. Results

The results are presented in two parts. First, the within-group pretest-posttest comparisons and the robust between-group analysis for need for cognition are reported. Second, the corresponding findings for the educational belief subscales are presented. Within-group findings are reported using the relevant parametric or non-parametric analyses. The results are presented as exploratory analyses.

Levene’s test indicated that the assumption of homogeneity of variances was met, F(1, 26) = 1.980, *p* = 0.171. As shown in Table 2, NFC scores increased significantly from pretest to posttest in the experimental group, t(12) = −4.37, *p* < 0.001, d = −1.21. The change in the control group was not statistically significant, t(14) = −1.22, *p* = 0.240, d = −0.32. Using the robust rank-based ANCOVA procedure and controlling for the corresponding pretest scores, no statistically significant difference was found between the experimental and control groups in posttest NFC scores, t(26) = −0.173, *p* = 0.864, η_p_^2^ = 0.001, 95% CI [−2.131, 1.799].

Levene’s tests indicated that the homogeneity of variance assumption was met for all educational belief subscales (all *p* > 0.05). As shown in Table 3, progressivism scores increased significantly from pretest to posttest in the experimental group, t(12) = −3.84, *p* = 0.002, d = −1.06. No statistically significant within-group changes were found for the other educational belief subscales in the experimental group or for any educational belief subscale in the control group. Using the same robust rank-based ANCOVA procedure and controlling for the corresponding pretest scores, no statistically significant differences were found between the experimental and control groups in the posttest scores of any educational belief subscale. Because these subscale analyses were conducted separately across multiple outcomes and were not adjusted for multiple comparisons, they were treated as exploratory and interpreted cautiously.

## 5. Discussion, Recommendations, and Conclusions

This research examined the associations of the P4TEduP with changes in teachers’ educational beliefs and NFC. Across the analyses, effect sizes were reported for all inferential comparisons. Large effect sizes were observed for the experimental group’s pretest-posttest changes in NFC and progressivism. However, after adjustment for the corresponding pretest scores, no statistically significant differences were found between the experimental and control groups in posttest NFC scores or any educational belief subscale. Accordingly, the adjusted between-group findings do not provide confirmatory evidence that the P4TEduP changed teachers’ NFC or educational beliefs in this sample.

Regarding the first sub-aim, NFC increased significantly from pretest to posttest within the experimental group. The within-group NFC finding can be considered in relation to Cacioppo et al.’s (1996) view that NFC reflects a motivational tendency toward cognitive engagement. Other studies support this emphasis as well. For example, in a quasi-experimental study with 12th graders, Ku et al. (2014) examined the effects of merging direct and inquiry-based critical thinking instruction. Although the intervention spanned only six classes, it improved NFC, and students with initially low NFC benefited more from inquiry-based education than their peers with high NFC. Kilgo et al. (2015) found that high-impact research, collaborative learning, and active learning improved NFC among university students. Similarly, Loes and An (2021) reported that collaborative teaching positively affected students’ NFC, particularly when supported by high-level, integrative, and reflective learning activities. Castle (2014) also reported a beneficial impact of collaborative learning on first-year college students’ NFC. These studies, which have a relevant pre-test–post-test group context, suggest that learning environments involving inquiry, collaboration, and reflective engagement may be associated with higher NFC. However, all were conducted in youth and young adult groups.

Philosophical inquiry intends to cultivate teachers’ inquiry and curiosity through discussion and offers possibilities for profound and reflective thinking, including metacognitive development (Bush, 2017). In this respect, the P4T and community of inquiry literature provides a relevant conceptual background for interpreting the NFC findings of the present study. In South Africa, L. Green and Condy (2016) explored how prospective teachers viewed Lipman’s community of inquiry pedagogy, to which these teachers said that a community of inquiry promotes active and critical thinking, reasoning and language skills, openness to other ideas, cooperation, and mutual respect. Similarly, Brubaker (2012) examined how pre-service teachers negotiate authority through a classroom community of inquiry: the community of inquiry’s collaborative discourse and deliberation fostered interdependent connections, active engagement, critical thinking and problem-solving, rather than personal disputes. That is, pre-service teachers recognised the need for an open communication environment to improve classroom comprehension and care, and that this helps them develop as future teachers. Furthermore, Bath et al. (2014) created a community of inquiry in a British university’s associate degree preparatory class to develop students’ critical thinking skills and found that it significantly impacted teachers’ thinking processes. Thus, the preceding studies provide direct NFC-related evidence at pretest, whereas the P4T literature helps explain the pedagogical features of philosophical inquiry that may be relevant to the observed within-group NFC pattern at posttest.

The critical task of philosophy is to problematise/question values, beliefs, and socially accepted ideas. In addition, it is the creative task of philosophy to prepare the conditions for identifying alternatives to any accepted idea or order (Kohan, 2015, p. 45). The P4TEduP aims to draw on both these tasks of philosophy while giving way for teachers to problematise various aspects of education in a community of inquiry context. For this purpose, the philosophical inquiry stimuli in the programme included important concepts/topics based on contemporary educational philosophies such as progressivism, reconstructionalism and existentionalism (Ornstein & Hunkins, 2012/2016). These concepts were used to stimulate teachers’ reflection on educational assumptions and belief systems. At the same time, given the absence of statistically significant adjusted between-group differences, these interpretive connections should be considered tentative and primarily useful for framing future empirical work.

Regarding the second sub-aim, the findings showed a significant pretest–posttest increase in the progressivism subscale within the experimental group, with a large effect size. In contrast, no statistically significant difference was found between the experimental and control groups for progressivism or any other educational belief subscale. This result can be considered alongside previous P4C and P4T studies. In particular, the philosophical inquiry activities conducted by Orchard et al. (2016) in a P4T workshop reported significant findings. Orchard et al. (2016) underpinned the social contributions of the community of inquiry membership and activities to teachers’ reasoning concerning ethical dilemmas (e.g., equality and justice in education) and the development of their collaborative and critical thinking skills. In his study with South African teachers, Roberts (2006) emphasised that the community of inquiry membership helps teachers re-evaluate their classroom actions and student relationships. Scholl (2014) found that a P4C-based programme had profound effects on teachers’ personal and professional selves, changing their locus of control and beliefs about students’ knowledge and abilities. Similarly, the experimental study by Scholl et al. (2016) showed that philosophical inquiry was effective in changing teachers’ pedagogies and the hegemonic classroom structure. Nevertheless, in the present study, this broader literature should be linked to the findings with caution, because the adjusted between-group analyses did not demonstrate statistically significant differences on any educational belief subscale.

Related P4C and P4T research also extends to pre-service teacher education. There are some studies depicting how the community of inquiry effectively fosters an epistemic perspective and student-centered educational understanding, discussion, and inquiry skills (Brownlee et al., 2014; Bush, 2017; Demissie, 2015), and reflection on high-quality teaching (Curtis, 2015). Akın Gökkuş and Aydın (2025) found that as teachers’ epistemological beliefs about learning increased, their evaluations of educational programmes and willingness to teach also increased. Within this body of work, the present study examines a P4T-based programme in an in-service teacher professional development context and reports a significant within-group increase from pretest to posttest in the progressivism subscale, although this finding was not supported by a statistically significant between-group difference. In light of this literature and the present findings, further examination of P4T-based workshops and seminars may warrant consideration as possible opportunities to support teachers’ reflection on educational philosophies and assumptions within in-service teacher education.

In conclusion, although statistically significant pretest-to-posttest increases in NFC and progressivism were observed in the experimental group, no statistically significant between-group differences were found. Building on Orchard et al.’s (2016) view of philosophical inquiry as a collaborative and democratic professional space, programmes such as the P4TEduP may offer teachers opportunities to discuss their professional experiences, educational assumptions, and concerns. These spaces may help teachers reflect on tensions between educational theory and practice and consider different perspectives within a community of inquiry. Overall, the within-group findings indicate patterns that may be examined in future research, whereas the present between-group evidence does not provide conclusive support for the view that the P4TEduP changed teachers’ NFC or educational beliefs. Accordingly, the study’s contribution is best understood as preliminary empirical and practical information that can guide future, adequately powered research, rather than as a theoretical advance or evidence of programme effectiveness.

## 6. Limitations

The quasi-experimental design used in this study means that the findings should be regarded as preliminary and exploratory rather than as strong causal evidence. Because there was no random assignment, baseline differences between the groups could not be fully controlled. Participation in the P4TEduP sessions was voluntary, and teachers were not randomly assigned to groups. Therefore, self-selection may have influenced the observed changes. A pretest was administered to assess initial group equivalence and mitigate this issue, but it cannot entirely eliminate pre-existing disparities. The study also involved a small sample of 28 teachers from a single school, which limits the generalizability of the findings. The sample was imbalanced by gender, which should be considered when interpreting the group comparisons. Future research should use stronger experimental designs with random assignment, larger multi-site samples, and, where feasible, an active control condition.

Another limitation of this study is that implementation fidelity was not assessed by an independent observer during the teacher intervention. Because the philosophical inquiry sessions were authentic and interactive, an independent observer could have influenced how participants expressed their thoughts and interacted within the community of inquiry. Nevertheless, the sessions followed predetermined rules, stages, and a structured inquiry process. Although the facilitator’s role in philosophical inquiry sessions does not involve directing the inquiry and the research findings were not based on a qualitative analysis of these sessions, possible researcher-facilitator effects should be considered as a potential limitation. Since the researcher contributed to the programme’s development and facilitated the sessions, care was taken to distinguish the researcher’s role from the facilitator’s role and to remain within that role.

The within-group findings for NFC and progressivism should be interpreted cautiously. The experimental group showed statistically significant pretest-posttest increases in NFC and the progressivism subscale, with large effect sizes, but no statistically significant between-group differences were found for either measure. The progressivism finding was limited to a single self-reported educational belief subscale, and initial scores were already high, suggesting a possible ceiling effect. These increases should be viewed as limited in practical significance because of the small sample size, the self-report nature of the measurement, and the absence of follow-up data.

Teachers from various educational levels and subject areas participated in the study, which likely enriched the discussions within the communities of inquiry. At the same time, this may have introduced limitations due to differences in professional language and context. Future studies could benefit from more homogeneous sampling by education level or subject area to better explore this factor. Furthermore, the control group did not receive an alternative active professional development activity during the main study period. Therefore, the specific contribution of philosophical inquiry cannot be fully separated from possible effects of additional attention, contact time, novelty, or participant expectations.

## 7. Preliminary Empirical and Practical Contributions

The present study contributes to the literature primarily in empirical and practical terms. Specifically, it adapts a training programme based on the P4T approach to an in-service teacher education context, whereas much of the prior literature has focused on pre-service teachers. It also adds preliminary empirical evidence on the possible role of philosophical inquiry in teacher professional development by implementing a programme for teachers that engages several sub-branches of philosophy. However, the study neither proposes nor directly tests a new theoretical model. Therefore, its contribution should be understood as a preliminary empirical and practical contribution that may inform future theory development and larger-scale studies.

The findings also offer preliminary practical implications for educational researchers and practitioners regarding the possible integration of philosophical inquiry into pre-service and in-service teacher education programmes. In this regard, philosophical inquiry may offer teachers opportunities to engage in effortful thinking and reflect on their educational beliefs. At the same time, because the adjusted between-group analyses did not yield statistically significant differences, these implications should be interpreted cautiously. The within-group patterns observed for NFC and progressivism may serve as an initial basis for future research and programme development rather than as confirmatory evidence of effectiveness.

## Figures and Tables

**Table 1:** Participants’ characteristics.

		Experimental Group		Control Group		Total	
		*n*	%	*n*	%	*n*	%
Gender	Women	3	23.1	10	66.7	13	46.4
	Men	10	76.9	5	33.3	15	53.6
Teaching field	Preschool Education	1	7.7	1	6.7	2	7.1
	Classroom Teaching	3	23.1	4	26.7	7	25.0
	English	2	15.4	4	26.7	6	21.4
	Physical Education and Sports	1	7.7	1	6.7	2	7.1
	Music	2	15.4			2	7.1
	Visual Art	1	7.7			1	3.6
	Turkish	1	7.7			1	3.6
	Social Studies	1	7.7			1	3.6
	Mathematics			2	13.3	2	7.1
	Turkish Language and Literature	1	7.7	1	6.7	2	7.1
	Psychological Counselling and Guidance			2	13.3	2	7.1
School level	Preschool	1	7.7	1	6.7	2	7.1
	Primary school	5	38.5	8	53.3	13	46.4
	Middle school	5	38.5	4	26.7	9	32.1
	High school	2	15.4	2	13.3	4	14.3
Total		13	100.0	15	100.0	28	100.0

**Table 2:** Paired-samples *t*-test results for within-group comparisons and HC3-robust rank-based residualised ANCOVA results for need for cognition scores.

Groups	*n*	Pretest		Posttest		*t*	*p*	d	Robust *t*	*p*	η_p_^2^	95% CI
		X^-^	SD	X^-^	SD							
**Experimental**	13	67.46	9.39	77.31	10.05	−4.37	<0.001 *	−1.21	−0.173	0.864	0.001	[−2.131, 1.799]
**Control**	15	67.87	11.43	68.87	10.49	−1.22	0.240	−0.32				

* *p* < 0.05.

**Table 3:** Paired-samples *t*-test and Wilcoxon signed-rank test results for within-group comparisons and HC3-robust rank-based residualised ANCOVA results for educational belief subscale scores.

Dimension	Groups	*n*	Pretest		Posttest		*t/z*	*p*	d/r	Robust *t*	*p*	η_p_^2^	95% CI
			X^-^	SD	X^-^	SD							
**Progressivism**	Experimental	13	4.56	0.42	4.73	0.41	−3.84	0.002 *	−1.06	−0.354	0.726	0.005	[−2.592, 1.831]
	Control	15	4.70	0.28	4.75	0.36	−0.89 ^a^	0.370	0.23				
**Existentionalism**	Experimental	13	4.65	0.39	4.73	0.41	−1.10	0.290	−0.30	−0.286	0.778	0.003	[−2.426, 1.834]
	Control	15	4.83	0.29	4.75	0.36	−1.45 ^a^	0.150	0.37				
**Reconstructionalism**	Experimental	13	4.05	0.80	4.11	0.56	−0.26	0.800	−0.07	−0.224	0.824	0.002	[−2.548, 2.047]
	Control	15	4.21	0.56	4.03	0.81	1.45	0.170	0.37				
**Perennialism**	Experimental	13	3.89	0.60	3.95	0.54	−0.32	0.760	−0.09	−0.380	0.707	0.006	[−3.629, 2.497]
	Control	15	3.98	0.85	4.08	0.90	−0.51 ^a^	0.610	0.13				
**Essentialism**	Experimental	13	1.92	0.87	2.17	0.94	−1.92	0.080	−0.53	−0.160	0.874	0.001	[−1.825, 1.563]
	Control	15	1.99	0.99	2.19	0.96	−1.54	0.140	−0.40				

^a^ Wilcoxon signed-rank test. All other within-group comparisons were conducted using paired-samples *t*-tests. * *p* < 0.05.

## Data Availability

The data presented in this study are available on reasonable request from the corresponding author. The data are not publicly available due to participant privacy and ethical restrictions.

## Abbreviations

The following abbreviations are used in this manuscript:

P4C	Philosophy for Children
P4T	Philosophy for Teachers
P4TEduP	Philosophy for Teachers Education Program
NFC	Need for Cognition
